# Decoding plant metabolism

**DOI:** 10.1093/plphys/kiag306

**Published:** 2026-05-29

**Authors:** Alain Goossens, Sibongile Mafu, Yang Zhang

**Affiliations:** Department of Plant Biotechnology and Bioinformatics, Ghent University, Technologiepark 71, Ghent 9052, Belgium; VIB Center for Plant Systems Biology, Technologiepark 71, Ghent 9052, Belgium; Department of Botany and Zoology, Stellenbosch University, Merriman Avenue, Matieland 7600, South Africa; Department of Biochemistry and Molecular Biology, University of Massachusetts Amherst, 240 Thatcher Way, Amherst, MA 01003, United States; Key Laboratory of Bio-Resource and Eco-Environment of Ministry of Education, College of Life Sciences, Sichuan University, No. 29, Wangjiang Road, Wuhou District, Chengdu, Sichuan 610065, China

Plants produce a remarkable diversity of metabolites that underpin plant growth, development, stress responses, and communication, while also serving as essential resources for humans. Understanding the complex metabolism, regulatory networks, and their functions has long been a central challenge in plant biology. Recent advances in technologies facilitate the discoveries and innovations. This collection brings together studies that illuminate both primary and secondary metabolism and regulation, explore their functional roles, and highlight cutting-edge technologies that accelerate our ability to decode and engineer plant metabolism. Eight updates and three topical reviews synthesize key advances in plant metabolism. Additionally, over 20 research articles in this collection shed light on the biosynthesis, regulation, diverse functions, and engineering of plant metabolites.

## Plant primary metabolism—core mechanisms, regulatory networks, and agricultural implications

Plant primary metabolism serves as the foundation for plant growth, development, environmental adaptation, and material synthesis, which permeates the entire life cycle of plants. It provides all organisms with essential energy, carbon skeletons, and nutrients. Moreover, plant primary metabolism possesses its own unique regulatory mechanisms that are tailored to the specific physiological needs of plants. It is not only closely associated with specialized metabolism, hormone signaling, and environmental responses but also acts as a core research field for deciphering plant life activities and improving crop quality and stress resistance. In-depth exploration of the regulatory mechanisms of plant primary metabolism is therefore of great significance for understanding plant physiological metabolic networks and promoting sustainable agriculture.

Fàbregas et al. (2026) have synthesized the current understanding of bidirectional communication between hormonal signaling and primary metabolism. Their conceptual framework illustrates how metabolic status serves as a feedback loop to influence hormone perception, highlighting the translational potential of these insights for modern crop breeding. Translating these concepts to application, Lai et al (2026) have utilized a multiyear field experiment to investigate the water–nitrogen synergistic regulation of maize productivity. The study reveals that such synergies bolster antioxidant defenses and optimize the balance of carbon–nitrogen metabolites and phytohormones, as well as establish a robust empirical basis for precision fertigation strategies.

Regarding the intersection of light response and metabolism, Timm et al. (2025) have reevaluated the metabolic scope of photorespiration. Far from being a mere detoxification pathway for 2-phosphoglycolate, photorespiration is characterized as a core metabolic hub linked extensively to nitrogen assimilation. The authors emphasized synthetic biology approaches to reengineer photorespiratory efficiency, noting its significant potential for enhancing crop environmental adaptability.

Two investigations into metabolic temporal dynamics have yielded significant insights. Fan et al (2026) have examined the diurnal shifts in leaf dark respiration (Rdark) across C3 and C4 species. Their analysis has shown that Rdark is intrinsically coupled with the tricarboxylic acid cycle and amino acid interconversion. Crucially, the study has established that Rdark measured in daytime dark-adapted leaves is physiologically distinct from nighttime respiration, cautioning against the interchangeable use of these data points in metabolic modeling. From an evolutionary standpoint, El-Azaz et al. (2025) have reconstructed the history of aromatic amino acid biosynthesis. Their research into the enzymes DHS and TyrA has revealed that the metabolic “deregulation” of tyrosine biosynthesis precedes the emergence of the grass-specific lignin pathway. This temporal sequence highlights the coordinated evolution between ancestral primary metabolism and lineage-specific secondary metabolic innovations.

In addition, Lu et al. (2025) have utilized crystal structure analysis and kinetic assays to characterize β-substituted alanine synthase 4;1 (BSAS4;1) in common bean. They have discovered that benzoic and salicylic acids act as competitive inhibitors of BSAS4;1, thereby modulating cysteine synthesis. The enzyme's putative role in S-methylcysteine production introduces a fresh paradigm for biofortifying sulfur-containing amino acids in leguminous crops.

## Specialized metabolism—integrated networks shaping plant development and adaptation

Plants synthesize an extraordinary diversity of specialized metabolites that support development, environmental adaptation, and ecological interactions. Advances in genomics, metabolomics, and molecular genetics have revealed that specialized metabolism is governed by highly integrated, multilayered biosynthetic and regulatory networks that coordinate enzyme activity, transcriptional control, hormone signaling, redox balance, and developmental plasticity. Such integration shapes metabolic diversity across plant systems.

Recent advances in major classes of specialized metabolites are reviewed in 5 updates in this collection. Huang et al. (2025) have summarized late development in volatile phenylpropanoid and benzenoid metabolism, covering biosynthesis and regulation and highlighting biological mechanisms facilitating transport and emission. Zhu et al. (2025) have provided an integrated overview of flavonoid biosynthesis and regulation. They have highlighted how the rapid expansion of knowledge drives engineering advances of nutritional and functional attributes of flavonoids in foods for improving their therapeutic potential. Pruckner et al. (2025) have examined isoprenoid metabolism in eukaryotic phytoplankton, emphasizing the biochemical diversity, evolutionary differentiation, and biotechnological potential that these pathways present. Li et al. (2025) have highlighted the latest findings in unraveling the complex regulatory networks governing carotenoid and apocarotenoid metabolism in plants, provided new insights into plastid differentiation for carotenoid accumulation, and updated the discoveries and functions of bioactive apocarotenoids. Liu et al. (2025a) have presented a comprehensive update on cytochrome P450 monooxygenases, underscoring their modularity, evolutionary diversification, and central role as hubs coordinating specialized metabolic networks for the biosynthesis of important bioactive compounds and mediating physiological functions.

Plastid remodeling acts as a driver of pigment metabolism. Plastid differentiation represents a critical control point linking development and metabolism. Zheng et al (2025) have investigated the determinants of plastid fate, focusing on the transition from chloroplasts to chromoplast-like structures. Through overexpression of Mg-decheletase STAY-GREEN, a key enzyme in chlorophyll degradation, the authors have demonstrated that chromoplast differentiation does not strictly require enhanced carotenoid biosynthesis. Instead, chlorophyll degradation alone can initiate plastid remodeling, provided that existing carotenoid pools are maintained. This work highlights how plastid differentiation is primarily driven by metabolic events but integrates developmental cues, such as fruit ripening, with environmental signals, including darkness and senescence.

Hormone-mediated transcriptional regulation acts as a key organizing principle of specialized metabolism. In sweet cherry, Titeli et al. (2026) have identified PaWRKY57 and PaNAC29 as central regulators of fruit color and growth outcomes, linking abscisic acid signaling with flavonoid and volatile biosynthesis. The integrated physiological, biochemical, and transcriptomic analyses across tissues and developmental stages constitute a valuable resource for improvement of sweet cherry traits.

Ethylene signaling similarly integrates developmental regulation with metabolic quality. Wang et al. (2026) have elucidated the functional redundancy and divergence of 4 tomato ethylene insensitive like proteins (EILs), key regulators of ethylene signaling. They have demonstrated that EILs are functionally divergent but mediate fruit coloration through MYB12, a master regulator of flavonoid biosynthesis, and ZISO, a structural gene in the carotenoid pathway. This work mechanistically connects ethylene signaling with fruit ripening and metabolic quality traits.

Extending the role of ethylene beyond fruit development, Tripathi et al. (2025) have demonstrated that ethylene regulates the metabolism and radial distribution pattern of steroidal lactones, withanolides, in mature tuberous roots of *Withania somnifera* (Ashwagandha). Accumulation is mediated through the control of sterol Δ24 isomerase, thereby influencing withanolide distribution. Identification of this control node provides strategies enabling improvement of metabolic yield of this important medicinal compound.

These hormone-driven regulatory frameworks extend beyond development to mediate metabolic reprogramming under environmental stress conditions as illustrated by Lv et al. (2025). The researchers have identified SmDof32 as a regulator of drought tolerance in *Salvia miltiorrhiza* (danshen). SmDof32 activates jasmonic acid biosynthesis through SmLOX8, leading to the induction of SmRAS1 and accumulation of salvianolic acid, directly connecting stress perception, hormone signaling, and bioactive compound production.

At the interface of development and stress adaptation, phenylpropanoid and lignin biosynthesis further illustrate how metabolic flux is constrained by cellular homeostasis and redox balance. Wang et al. (2025) have examined the coordinated expression of phenylalanine ammonia lyase (PAL) and cinnamate 4 hydroxylase (C4H) during bamboo elongation. Using CRISPR based knockouts, they have uncovered complex feedback regulation mediated by trans cinnamic acid, involving both transcriptional and post translational control of PAL10 and compensatory activation of C4H under PAL suppression.

Zhu et al. (2026) have demonstrated that cytosolic ascorbate peroxidases function as coumarate 3 hydroxylases, directly integrating redox metabolism with lignin biosynthesis. Genetic analyses in Brachypodium and Populus have shown that loss of C3H/APX function reduces lignin content, alters monomer composition, elevates hydrogen peroxide levels, and impairs growth. Severe developmental defects observed in combined C3H/APX mutants have demonstrated how redox homeostasis is tightly integrated with phenylpropanoid metabolism and vascular development.

Collectively, these reviews and research articles reinforce specialized metabolism as an integrated and dynamically regulated component of plant biology, governed by interconnected transcriptional, hormonal, redox, and developmental processes. Identifying shared regulatory nodes across pathways and contexts not only advances conceptual understanding but is also essential for harnessing metabolic diversity to improve crop resilience, productivity, and quality.

## Metabolism—a central integrator of plant function

Plant metabolites are central to biological function, serving not only as substrates for metabolism but also as direct determinants of physiological outcomes. Beyond sustaining growth and development, many metabolites confer specific functions, enabling plants to sense, respond, and adapt to their environment. Increasingly, metabolites are recognized as regulators and signaling molecules that coordinate and mediate processes across growth, development, ecological interactions, and stress responses (Erb and Kliebenstein 2020). Their roles are often context-dependent, varying across cell types, developmental stages, and environmental conditions. As new roles continue to emerge, understanding how specific metabolites are linked to specific functions remains a central challenge in plant biology and a key opportunity for crop improvement.

Root-derived metabolites exemplify this integration. Uribe-Acosta et al. (2025) have described roots as metabolic architects of their microbiome, illustrating how root metabolites, from primary compounds to specialized molecules, act as selective forces that recruit beneficial microbes. This work positions root metabolites as a dynamic interface in plant–microbe interactions and highlights its potential for microbiome-informed crop improvement and sustainable agriculture.

Specialized metabolites form complex chemical repertoires, yet how they are deployed remains a central question. The Topic Review by Somssich et al. (2025) has synthesized decades of work in *Arabidopsis thaliana*, illustrating how plant metabolites such as glucosinolates, camalexin, and volatile terpenes function not merely as static products but as dynamic, spatially organized, and environmentally responsive chemical defense molecules. This frames specialized metabolism as an active system underlying plant survival strategy.

Isoprenoid metabolism further highlights the expanding functional landscape of plant metabolites. In their Update Review, Li et al. (2025) have discussed the expanding roles of carotenoids and their bioactive apocarotenoid derivatives as regulators and signaling molecules in growth regulation, stress responses, and symbiosis. Complementing this perspective, Hazra et al. (2025) have uncovered a previously unrecognized function of β-carotene derived apocarotenoids in prolonging seed longevity, acting through oxidative stress mitigation and engaging an aquaporin protein as part of the signaling pathway. Extending beyond land plants, Pruckner et al. (2025) have highlighted the remarkable metabolic diversity of isoprenoids in phytoplankton and for their roles in algal physiology. By using specialized metabolites such as sterol sulfates and the isoprenoid-derived domoic acid as examples, the authors have illustrated key unresolved questions for the ecological functions of isoprenoids in algal ecology.

Flavonoid metabolism shows how metabolic pathway activities are repurposed to regulate development and defense. Chen et al. (2026) have demonstrated that 2 flavonoid pathway genes, *GhCHS* and *GhDFR*, distinctly regulate cotton fiber development by balancing ROS homeostasis during fiber elongation and coordinating cellulose biosynthesis and wall thickening during secondary cell wall formation. In parallel, Yang et al. (2026) have reported species-specific UDP-glucosyltransferases, *VmUGT88A65* and *VmUGT88A66*, redirecting metabolic flux into flavonoid and lignin glycosides with root growth, thereby reinforcing disease resistance in tung trees. These examples highlight how flavonoid metabolic pathways are redeployed to specific functions.

Cytochrome P450 enzymes further link metabolic diversity to physiological function. The update by Liu et al. (2025a) has highlighted recent advances on how a subset of cytochrome P450 enzymes regulates the production of bioactive metabolites and their significant physiological function in modulating diverse aspects of plant growth, development, and stress adaptation via mechanisms including directly regulating fundamental biological processes and through regulating phytohormones and protective specialized metabolites.

Together, these contributions converge on a unifying theme: plant metabolism is a central integrator of function, dynamically coordinating development, defense, and environmental interactions. The emerging challenge is not to catalog metabolites, but to define their roles in controlling specific biological processes and physiology.

## Frontiers technologies—key in providing deeper insights into metabolic networks

Plants constantly perceive internal and external cues, which need to be addressed to safeguard growth, development, and fitness. Response to these cues typically involves selective modulation of specific metabolic pathways and networks, which in turn are governed by a plethora of molecular players that act and interact in complex, multilayered regulatory networks (Lacchini and Goossens 2020). Discovering and unraveling the metabolic pathways and their regulatory networks requires robust and sensitive tools and technology platforms, enabling the mapping and monitoring of metabolites, proteins, transcripts, and genes at distinct levels of resolution, range, and comprehensiveness. This is empowered by recent advances in omics, offering us the desired novel insights that will ultimately facilitate rational manipulation of plant metabolic processes. In this focus collection, some of these advances are being highlighted in 2 review articles and their impact exemplified in 2 research articles.

Rai et al. (2025) have presented an Update Review on the advances in plant metabolomics technologies and their impact on the field. Their review traces the evolution of plant metabolomics, starting from targeted analysis to the present high-throughput untargeted era of metabolomics. They have elaborated on metabolome analyses of responses to biotic and abiotic stress conditions, in the model plant Arabidopsis, in agronomic and horticultural crops, and in medicinal plants, and how these analyses unveiled roles of plant specialized metabolites or contributed toward the design of engineering strategies to improve plant traits. A notable effort of the authors is the identification and summary of over 500 open-source computational tools for analyzing metabolomics datasets, encompassing processes from mass feature extraction to metabolite annotation. Advances in metabolomics are also covered in the Topical Review by Ntelkis et al. (2025), more precisely in terms of increasing omics resolution to the single-cell level. Understanding where and when metabolites accumulate is essential for uncovering their function, biosynthesis, and regulation. This is particularly applicable to plant specialized metabolism, which is often compartmentalized at the cell-type level. The authors have listed and compared transcriptomics and metabolomics technologies that enable the direct visualization and quantification of metabolites and gene expression at a cellular resolution. They have exemplified how these platforms are currently used to investigate cell- and tissue-specific metabolism by describing case studies in several model and medicinal plant species. Finally, they have also presented an outlook on the transition from singleomics to multiomics approaches, which will enable a broader and deeper interrogation of molecular and biochemical processes at the cellular level.

Liu et al. (2025b) have generated a valuable dataset for the tomato research community, more precisely by constructing a comprehensive noncoding (nc) RNA-mediated metabolic regulatory network. This dataset encompasses >400 microRNAs (miRNAs) and ca 3,000 long ncRNAs (lncRNAs) from 3 tomato subgroups. In parallel, the authors have assessed the expression of these regulatory elements during domestication and integrated that with targeted accumulation profiles for 280 metabolites in multiple tomato tissues. Together, this enables the construction of an ncRNA-mediated metabolic regulatory network for tomato. The value of this dataset was demonstrated by the identification of an UDP-glycosyltransferase (UGT) as the target of miR172, validating the role of the miR172a-UGT module in the formation of glucosylated flavonoids.

## Metabolic engineering—advances in translating knowledge into applications

Plants are considered as the most sophisticated biofactories in nature, providing unique and invaluable sources of food, therapeutics, pesticides, biofuels, flavors, fragrances, etc. By engineering plant metabolism, the production of desired compounds can be increased, plant resilience improved, or the nutritional or commercial value of the plant species enhanced. Engineering plant metabolism is most often a very challenging endeavor because of the complexity of the regulatory and metabolic networks (Selma et al. 2023). Improvement in metabolic engineering capacities can come from (i) the development of different tools and strategies for rerouting metabolic pathways or rewiring regulatory circuits, and (ii) the discovery or increased understanding of (novel) biosynthetic pathways and regulatory mechanisms. The latter element benefits largely from the emerging frontiers technologies, some of which have been highlighted in the previous section, which are providing new and robust methods to dissect plant metabolism at an unprecedented resolution, scale, and speed. Likewise, the application of the emerging tools and technologies is no longer limited by the identity or class of the target metabolite(s) and, in the near future, neither the target plant species. This is also evident from this Focus collection.

Zhu et al. (2025) have provided an Update Review on the state-of-the-art in metabolic engineering of flavonoids. The demand for flavonoids, particularly for therapeutic uses, remains high, which has instigated researchers across the world to explore alternative synthetic routes. Accordingly, the past decade marked the design of performant microbial production systems for tens of different flavonoids, in both bacterial and yeast hosts. Equally, the plant chassis keeps having advantages for the (heterologous) synthesis of flavonoids, with a protagonist role for transcription factors as potent engineering tools and the development of purple tomatoes as a benchmark example for the current power of plant metabolic engineering. Pruckner et al. (2025) have reviewed the biotechnological opportunities in eukaryotic phytoplankton. They have discussed synthetic biology strategies to harness the metabolism of various microalga for the sustainable biosynthesis of a wide variety of high-value terpenoids, including monoterpenoids, diterpenoids, sesquiterpenoids, ketocarotenoids, and cannabinoids.

Finally, the Hiroshi Maeda group reports on a successful in plantaengineering effort. In El-Azaz et al., (2025), they have studied the evolution of aromatic amino acid (AAA) biosynthesis during the emergence of the tyrosine-derived lignin pathway uniquely found in grasses and closely related Poales. Based on the insights gained and grass AAA enzymes identified during this study, they have engineered levels of tyrosine and other AAAs in *Nicotiana benthamiana* leaves.
